# Comprehensive corrective exercise program improves ankle function in female athletes with limited weight-bearing ankle dorsiflexion: A randomized controlled trial

**DOI:** 10.1371/journal.pone.0312152

**Published:** 2024-10-31

**Authors:** Tahereh Sohrabi, Farzaneh Saki, Farzaneh Ramezani, Behdad Tahayori

**Affiliations:** 1 Department of Exercise Rehabilitation, Faculty of Sport Sciences, Bu-Ali Sina University, Hamedan, Iran; 2 Department of Physical Therapy, University of Saint Augustine for Health Sciences, Miami, FL, United States of America; Prince Sattam bin Abdulaziz University, SAUDI ARABIA

## Abstract

Limited ankle dorsiflexion range of motion is one of the most important risk factors for lower limb injury, which changes the biomechanics and the neuromuscular control of the lower limb muscles. This study aims to test the effectiveness of a comprehensive corrective exercise program (CCEP) on the range of motion, proprioception, dynamic balance, and muscle activation in female athletes with limited weight-bearing lunge ankle dorsiflexion range of motion. 30 female athletes aged 15 to 25 years with dorsiflexion under 34° were randomized to two groups. The intervention group (n = 15) received eight weeks of CCEP including soft tissue mobilization, joint mobilization, stretching, and strengthening, and the control (n = 15) group did not receive any intervention. range of motion, proprioception, dynamic balance, and muscle activation were assessed before and after the intervention. The training group showed clinically acceptable and statistically significant changes in ankle dorsiflexion range of motion (ES = 0.714), balance (ES = 0.423), and proprioception (ES = 0.253; P < 0.05). There were significant changes in the activity of the tibialis anterior and soleus muscles in the dynamic overhead squat test (descending and ascending phases) and the activity of the medial gastrocnemius in the descending phase decreased significantly (P < 0.05). No significant change was observed in the activity of the peroneus longus muscle (P > 0.05). The findings show that CCEP appears to be beneficial in increasing dorsiflexion range of motion, proprioception, balance, and decreasing ankle muscle activity among individuals with limited ankle dorsiflexion. Improving the dorsiflexion range of motion may be promising for reducing ankle sprain injury.

## Introduction

Limited ankle dorsiflexion range of motion (ROM) is recognized as an intrinsic risk factor for lower limb injuries [1]. Systematic reviews and prospective studies have identified a clear association between limited ankle dorsiflexion ROM and a heightened risk of knee pathology, Achilles tendinopathy, hamstring injury, and an increased risk of ankle sprains [2–6]. A prospective study reported that a decreased ankle dorsiflexion (with the least flexible range measured at 34°) was associated with a 2.5 times higher risk of tendinopathy [4]. Malliaras *et al*. [5] measured maximal dorsiflexion using a modified lunge and found that when dorsiflexion was less than 45°, it compromised lower extremity biomechanics and the ability to absorb load, which can be predictive of subsequent injury. Baumbach et al. [6] established the physiological lower limit of ankle dorsiflexion at 30° in the weight-bearing lunge test. They demonstrated that values below the 30° for ankle dorsiflexion can indicate impaired ankle function. This emphasizes the clinical importance of interventions targeting the improvement of dorsiflexion ROM as a preventive measure. Pathologies such as plantar flexor muscles tightness, decrease in the posterior glide of the talus in the mortise, and loss of accessory motion at the tibiofibular, subtalar, and midtarsal joints can all contribute to limited dorsiflexion ROM [7, 8].

The intervention usually used to improve ankle dorsiflexion ROM is stretching the gastrocnemius muscle complex [9]. Stretching interventions range from static stretching to proprioceptive neuromuscular facilitation techniques. The results of a study comparing four stretching techniques (static, agonist contraction, isometric contraction-relax, and hold-relax-agonist contraction) of the leg muscles in the sagittal plane showed that the ankle dorsiflexion ROM increased in all groups and no significant difference was observed between different groups. Therefore, the results showed that any type of stretching of the posterior muscles of the leg is effective in increasing ankle dorsiflexion ROM [10].

Non-contractile structures (joint capsule and ligaments) also play a role in joint ROM. When there are limitations in the talocrural (ankle) joint, full dorsiflexion cannot be performed. As such, a lack of joint mobilization can be observed when there are limitations in non-contractile structures [11, 12]. The results of previous studies showed that improving ankle joint mobilization, especially in the talocrural joint, improves the posterior glide of the talus in the ankle mortise, and as a result, increases the ankle dorsiflexion ROM and decreases injury. Self-mobilization of the talus joint in the posterior direction is a common intervention that is used to overcome limitations in capsular structures [11, 12].

Despite the supporting evidence for the benefits of stretching and mobilization, there is a lack of a standardized corrective exercise program. This study aims to fill that gap by investigating the effect of a comprehensive corrective exercise program (CCEP) on muscle activation, ROM, proprioception, and dynamic balance. We hypothesize that participants undergoing the CCEP will demonstrate significant improvements in ankle dorsiflexion ROM compared to those who do not receive the intervention. The findings are expected to contribute to the development of a standardized protocol for addressing limited ankle dorsiflexion, thereby aiding clinicians in the prevention and management of related lower limb injuries.

## Materials and methods

The Ethics Committee of the Bu-Ali Sina University of Hamedan (IR.BASU.REC.1400.042) approved this study. This study has been registered with the Iranian Registry of Clinical Trials (IRCT20190224042827N4) and follows the guidelines of the Declaration of Helsinki 2013. The athlete screening process began on November 12, 2022, and the post-test was conducted on March 6, 2023.

### Study setting and participants

**Fig 1:**
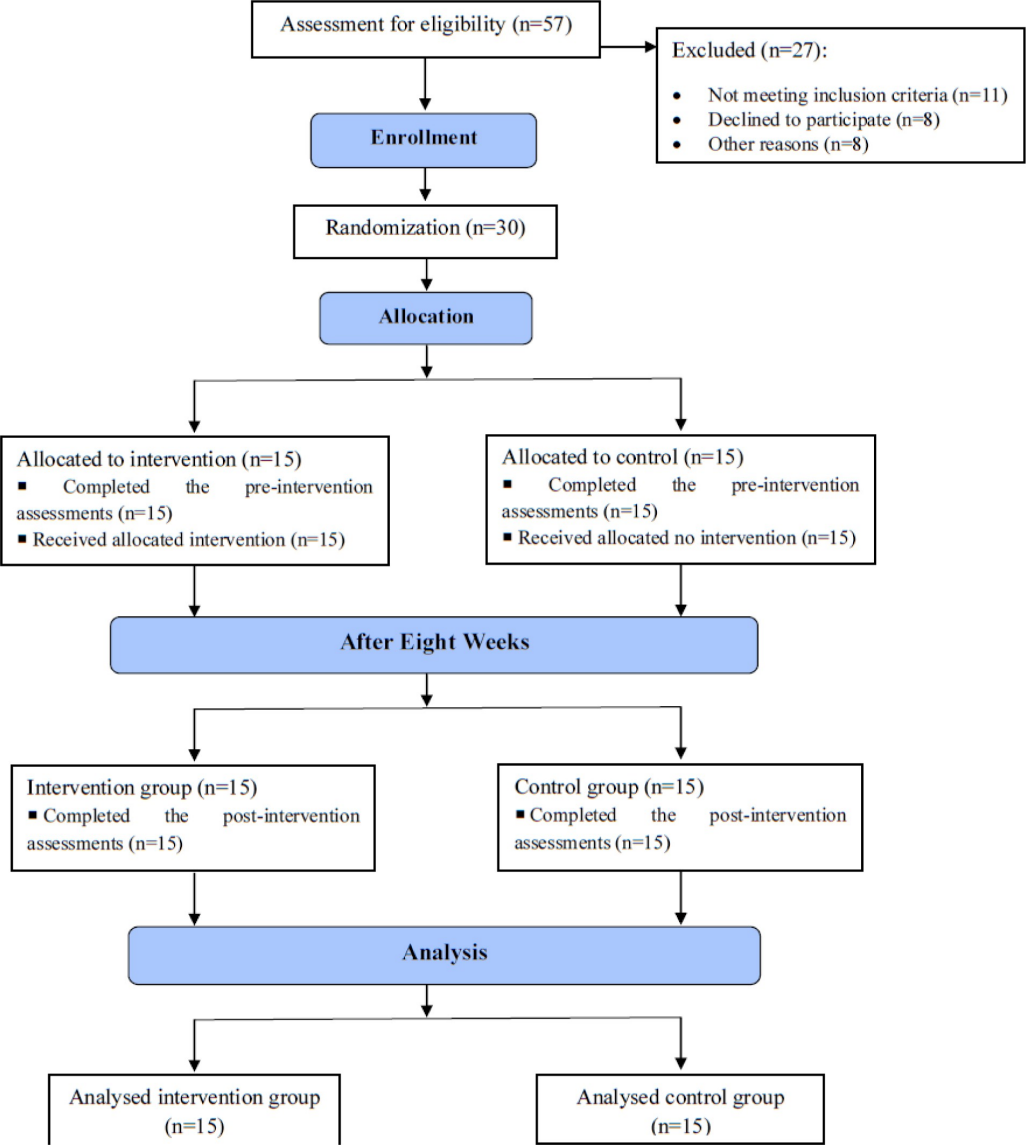
Study flowchart.

A total of 57 young women, ranging from 15 to 25 years old, who participated in track and field were gathered for the study through personal referrals and by distributing flyers at athletic clubs within the city of Hamedan. The inclusion criteria were: athletes with a decreased ankle dorsiflexion during weight-bearing lunge test (≤ 34°) [4, 13], training at least three sessions per week (60 minutes each session), and a body mass index of 20 to 25 kg/m^2^. The exclusion criteria were pain, history of lower extremity surgical procedure, any lower extremity injury within the past six months, and contraindications for Instrument-assisted soft tissue mobilization including a history of arteriosclerosis, thrombosis, embolism, severe varicose veins, acute phlebitis, cellulitis, synovitis, abscesses, skin infections, cancers, and acute inflammatory conditions (Fig 1) [14].

### Study design

This study was a single-blind, randomized controlled trial. The independent variable was the application of the CCEP, while the dependent variables included changes in ankle dorsiflexion ROM, proprioception, dynamic balance, and muscle activation. Using a single-blind design, the assessors responsible for measuring these dependent variables were unaware of the group assignments, ensuring they did not know which participants had received the intervention. Participants were informed about the study procedure and Written informed consent to participate was obtained from the parents or legal guardians of all participants prior to their involvement in the study. The sample size was determined using the G-Power software® (version 3.1.9.2; Franz Faul, University Kiel, Germany). Based on a 0.85 confidence level, a 0.05 alpha level, and a medium effect size of 0.50, the minimum sample size was determined to be 12. Considering the possibility of dropping out for subjects, the sample size was 15 individuals per group. Using Random Number Generator software® v1.4, participants were randomly assigned to one of the groups. Allocation concealment was done by sequentially numbered opaque sealed envelopes technique. The size of each group was as follows: training group (n = 15), and no training group (n = 15).

### Outcomes

All participants were asked to fill out a questionnaire that included age, height, weight, and history of sports activity (Table 2). Closed kinetic chain (CKC) ROM, ankle proprioception, dynamic balance, and electromyography (EMG) activity of the ankle muscles were measured before and after the eight-week program. All measurements were performed three times for the dominant leg with a 30-second rest interval. The dominant leg was defined as the leg participants would use to kick a ball [15]. Average scores were calculated and used for data analysis.

### Closed kinetic chain ROM

**Fig 2:**
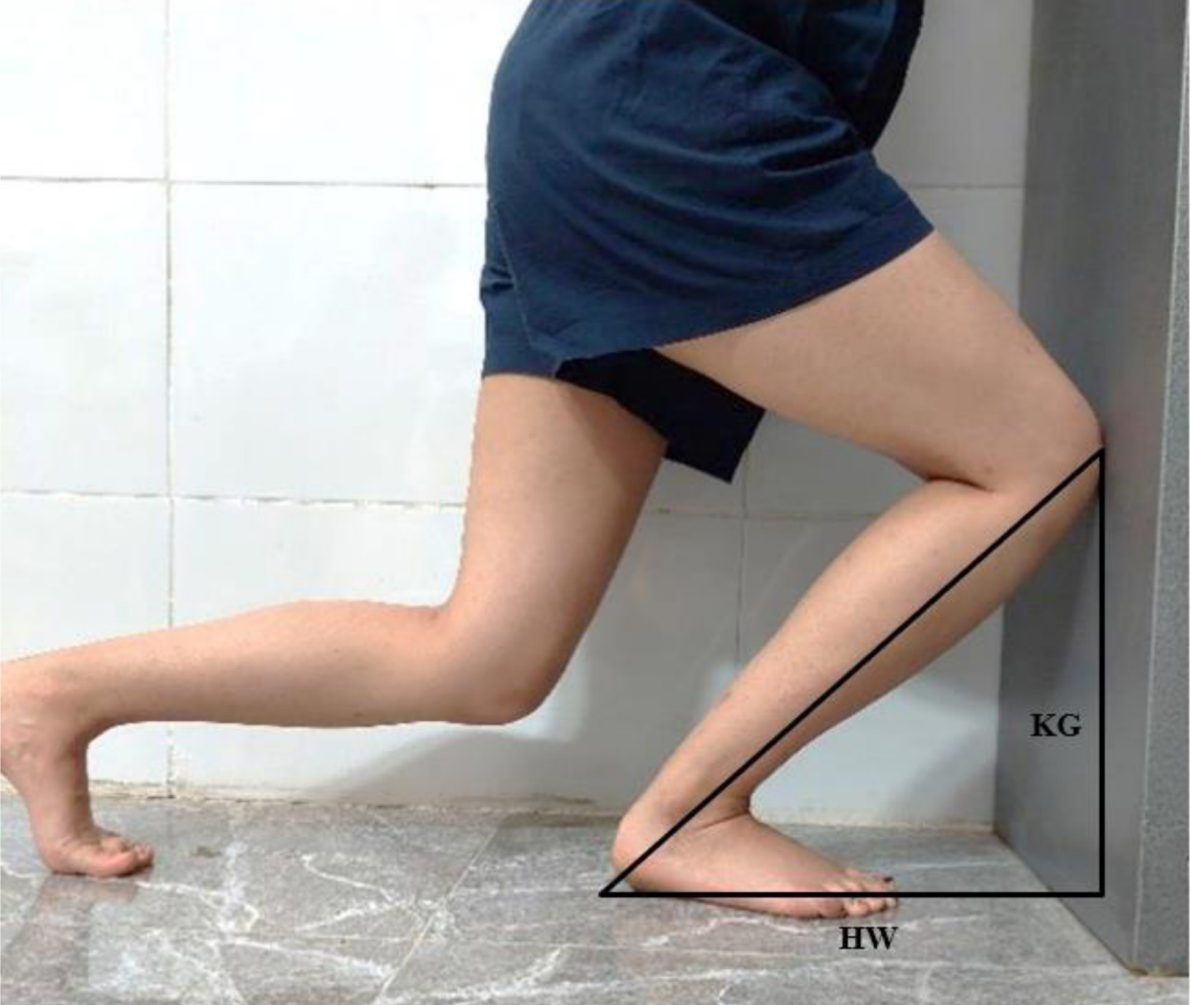
Measurement of ankle joint dorsiflexion ROM using trigonometric theory.

The trigonometric technique measured ankle dorsiflexion ROM in CKC and weight-bearing position (ICC = 0.97) [16]. In this method, participants were instructed to assume a lunge position against a wall while maintaining heel contact with the ground. Ankle dorsiflexion ROM was calculated through trigonometry theory with the distance between the heel to the wall (HW) and knee to ground (KG). At the same time, the participant performed a weight-bearing lunge test (Fig 2) [17].

Ankle dorsiflexion ROM = 90° − Arctangent [KG/HW]

### Ankle proprioception

**Fig 3:**
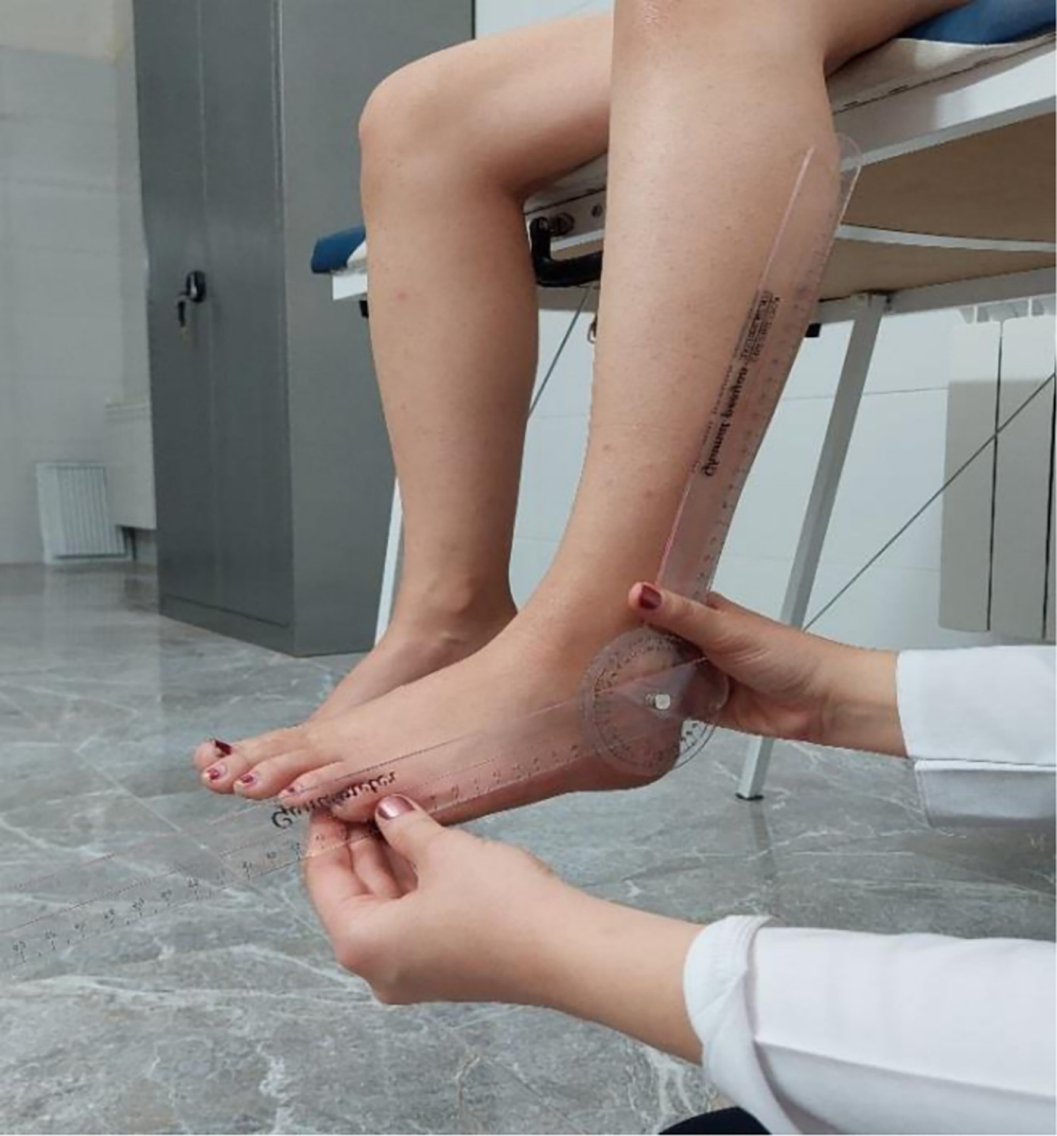
Measurement of the ankle proprioception.

Participants sat on an examination table with their legs hanging freely, without any ground contact. The passive-active method, known for its efficacy in assessing joint position sense error, was employed To familiarize participants with the target angle, the examiner passively positioned the ankle in 20° of plantar flexion three times, holding each position for five to ten seconds [18]. Afterward, the ankle was returned to neutral, and participants were asked to slowly reposition it to the target angle. Ankle proprioception was measured using a goniometer (ICC = 0.86–0.83) [19], and the mean error was recorded as the absolute repositioning error [20]. During the assessment, participants were blindfolded to eliminate visual input, and noise-canceling headphones were used to block auditory cues. This ensured that the evaluation relied solely on proprioceptive feedback from the ankle joint, providing a more accurate measure of joint position sense error (Fig 3).

### Dynamic balance

**Fig 4:**
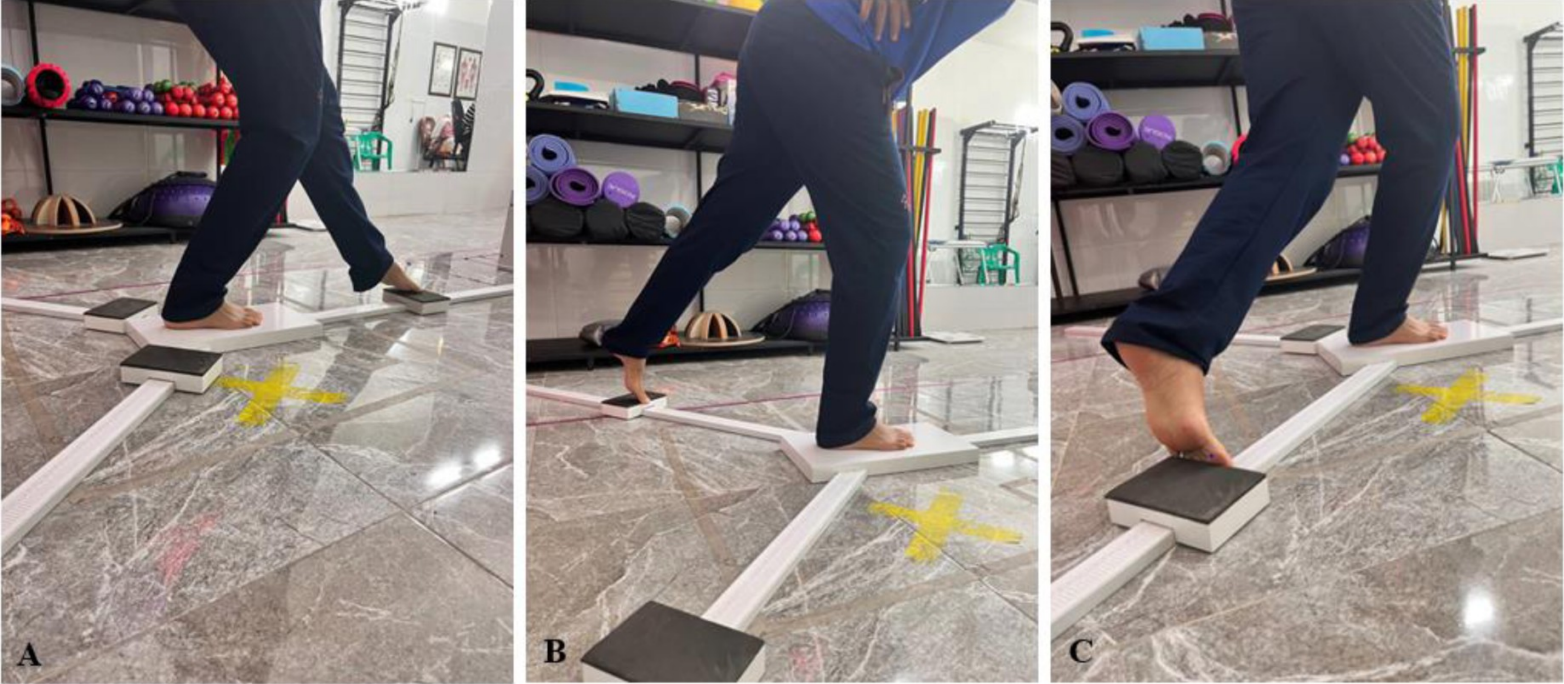
Measurement of the Y-Balance test. A; antrior, B; posterolateral, and C; posteromedial.

A standard Y-Balance test kit was used to assess dynamic balance (ICC = 0.85) [21]. The Y-Balance test has a significant relationship with leg length; to normalize data the leg length information was measured from the anterior superior iliac spine to the inner malleolus while the participant was in the supine position. The Y-Balance test was conducted on the involved limb only. Before starting the assessment, participants received detailed instructions about how to perform the test and were allowed to practice three experimental practice attempts, following a five-minute interval, the main test was conducted. To perform the test participants were asked to remove their footwear, and stand on the center platform, with their hands firmly placed on their hips. Participants were then instructed to slide the first box forward as far as possible with their free (non-limited) foot and return to the starting upright position. They were asked to repeat this with the same foot for a total of three successful reaches. After they completed three successful reaches, they would then progress to the next test direction (i.e. posterolateral, and posteromedial). The maximum distance reached was measured and recorded in three directions: anterior, postero-medial, and postero-lateral in the last three replications [22, 23]. The composite score was calculated by taking the sum of 3 reach directions divided by 3 times the limb length and then multiplied by 100 [22]. The composite score was taken exclusively for the limb affected by the condition, incorporating data from all three directional reaches of the test (Fig 4).

### Electromyography

Muscle activity was recorded by four wireless surface EMG sensors (Biometrics Ltd, Newport, UK). These EMG sensors were placed on tibialis anterior, peroneus longus, medial gastrocnemius, and soleus. Data were obtained from the leg with limited dorsiflexion. EMG data were collected at a 1000 HZ sampling rate. Surface electrodes (LE230, 42 × 24 × 14 mm, center-to-center electrode distance of two cm) were placed on the bulk of the muscles aligned with the muscle fibers, as recommended by established literature [24–26].

**Fig 5:**
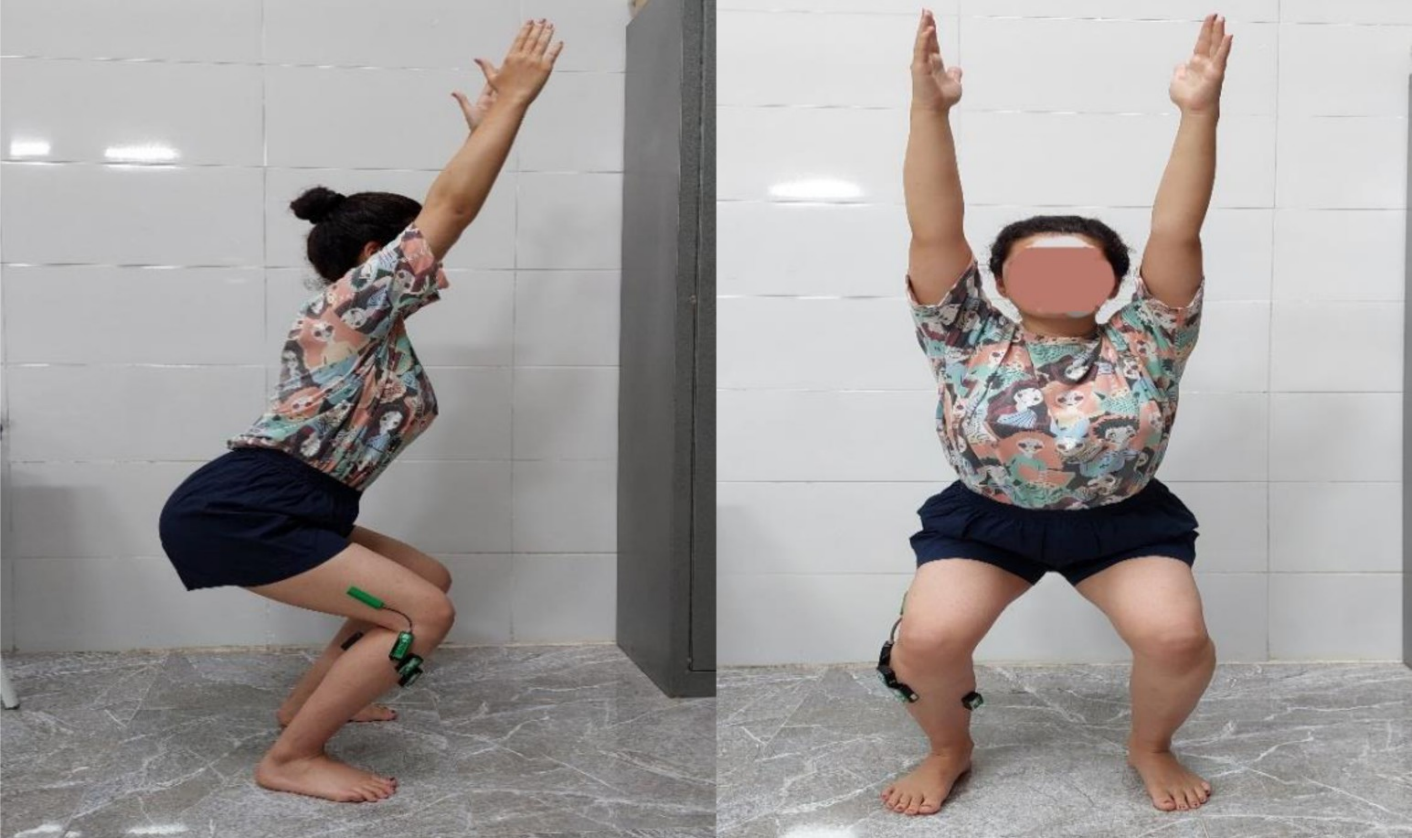
Measurement of the overhead squat.

After placing the electrodes on the muscle, each participant was asked to do the overhead squat. Participant stood up, with their feet roughly shoulder-distance apart, toes angled slightly outward, and placed their hand next to their ears. They took a deep breath and braced their core to help keep their spine stable. This test was measured with knee flexion angle at 80° (an electrogoniometer determined this angle) and with a 30-beat metronome, full cycle: five beats (two down beats, one pause beat, two up beats) three times (Fig 5).

**Fig 6:**
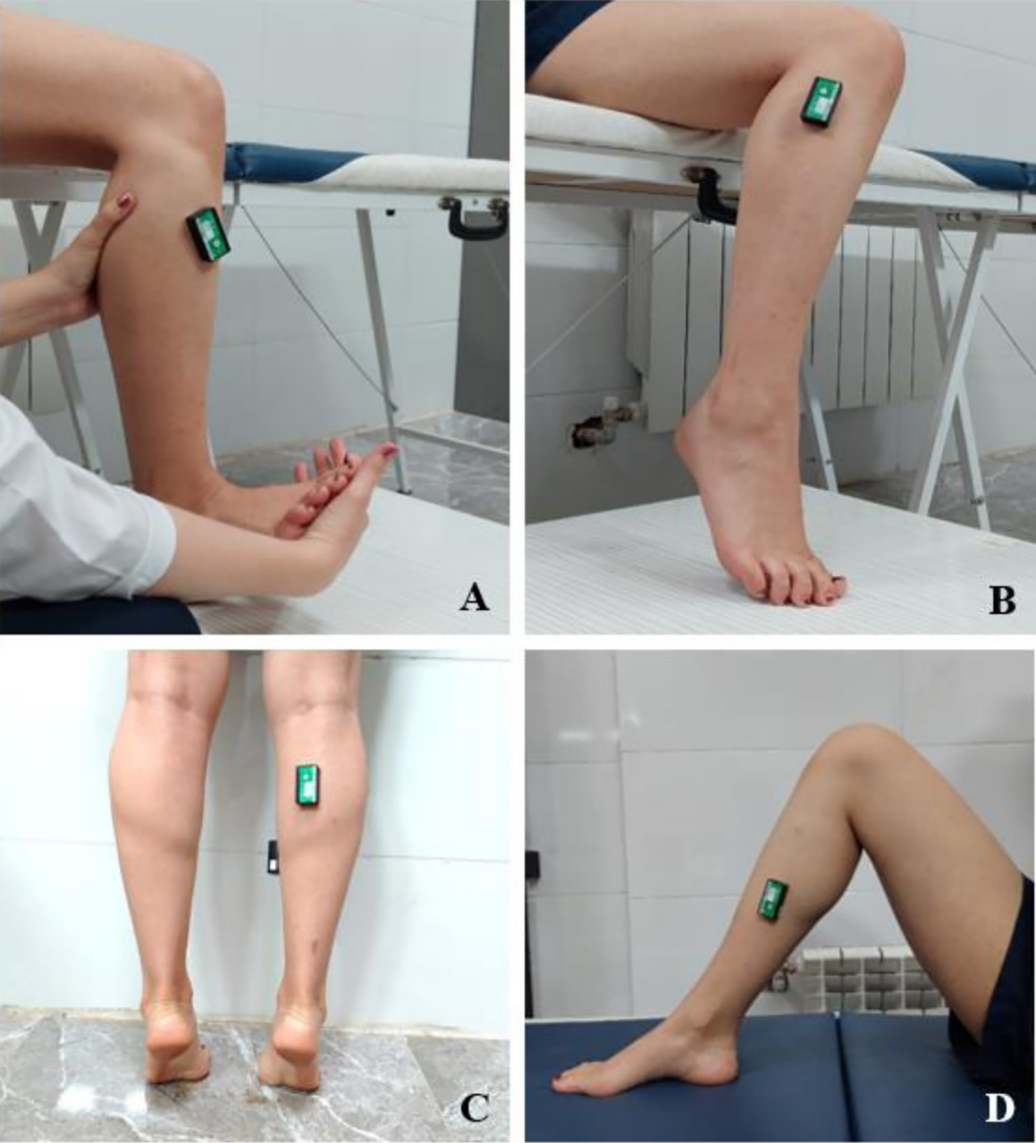
EMG electrode location. A; tibialis anterior, B; peroneus longus, C; medial gastrocnemius, and D; soleus.

Maximum voluntary isometric contraction (MVIC) was performed and recorded for each muscle separately after the functional task. Each isometric contraction was held for six seconds and repeated three times with one-minute rest between the contractions. The average of these three trials was calculated for each muscle to establish a baseline for normalization. Verbal encouragement was used to provoke the participants’ maximum effort. Participants carried out maximal contraction following the protocol suggested in [26–28]. EMG data were analyzed in Matlab® Software (MathWorks, Inc, Natick USA). A 10–500 Hz band-pass filter was applied to the raw signal. Occasionally, there was a need to apply a 50-HZ notch filter when mains noise was present. Then, a 50-datapoint moving-window root means square of the filtered data was calculated for the ascending and descending phases of the squat (Fig 6). Muscle activity percentage was calculated by dividing the activity of each muscle by the MVIC value and multiplying the result by 100.

### Intervention

The CCEP has been adopted from previous studies [12, 14]. The protocol includes warm-up, soft tissue mobilization, joint mobilization, stretching, and strengthening (Table 1) [29]. The protocol was performed 30 minutes a day for eight weeks (three sessions per week; 24 sessions). The training group performed the CCEP, while the control group did not engage in any exercise treatment during the study period. However, following the eight-week study period, the control group also received the exercise treatment. At first, participants performed a 15-minute warm-up protocol focusing on the lower extremity muscles. Then, the participants lay prone on an examination table while the hip was in full extension, the knee in partial flexion, and the ankle in plantar flexion. The gastrocnemius muscle, Achilles tendon, and plantar fascia were smeared with Graston Technique (GT) emollient. At first, a sweeping stroke was used to scan the adhesions for one minute with the GT instrument. Then, four minutes were used to focus on adhesions for the remaining. For the first six sessions, GT instruments were used at a 30° to 45° angle while moderate pressure was maintained, and sweeping, fanning, and scooping strokes were used in all directions. From the seventh to twelfth sessions, the GT instruments were used at about 60° angle and with more pressure. After the twelfth session, a foam roller was used for 30 seconds to release the gastrosoleus muscles as self-mobilization. Following the soft tissue mobilization participants were asked to perform stretches of the gastrosoleus muscles and mobilize the talocrural joint in a standing position with the use of a strap. To do this movement, participants positioned the knee of the leg being trained approximately 12–14 cm ahead of the non-training leg. A strap secured to a stable object was positioned around the front part of the ankle joint. Participants were then guided to gently move the knee of the trailing leg forward into dorsiflexion, ensuring the movement was pain-free. Maintaining contact between the heel of the trailing leg and the ground is important throughout the exercise. Finally, the participant performs strength exercises as part of the CCEP. The standing heel-toe raises exercise progressed from double leg to single leg and from straight knee to bent knee. Additionally, the exercises were performed on the ground and then continued on the step to increase the intensity. This program was administered by certified physical therapists with specialized training in sports injury prevention and rehabilitation. They are experts in GT and joint mobilization. Their expertise ensured the correct application of these techniques and the tailoring of the intervention to each participant’s needs. The CCEP program was performed face-to-face at the Bu-Ali Sina University sports therapy facility. We customized the exercises for each athlete, based on individual’s needs and progress throughout the intervention. The initial phase focused on familiarizing participants with the correct form and execution of the exercises. As participants demonstrated proficiency and improved strength and flexibility, the intensity and complexity of the exercises were progressively increased. This included variations in the number of sets, repetitions, and resistance used, to continually challenge the muscles and prevent plateauing. The progression was carefully monitored and adjusted based on individual responses to ensure safety and optimal benefit from the program. Adherence to the program was successful because it offered a variety of exercises and used ways to keep athletes engaged and doing the exercises correctly.

**Table 1:** Comprehensive corrective exercise program.

Program	Exercise	Protocol
Warm-up	Stationary bicycle	15 min
Soft tissue mobilization	Scan the calf for adhesions for 1 minute. Focus on adhesions for the remaining 4 minutes. During the first 6 sessions, instruments were held at a 30° to 45° angle while moderate pressure was maintained, and sweeping, fanning, and scooping strokes were used in all directions. In sessions 7 to 12, the treatment angle increased to 60° while also increasing the pressure maintained to increase the intensity of treatment.	5 min
	After the 12th session, a foam roller was used for the self-release of the calf muscles. The foam roller rested on the painful areas for the 30s.	5–8 min
Joint mobilization	Standing calf muscles stretch with strap for talocrural joint mobilization	5 times, holding each stretch for 30 seconds
Stretching	Calf muscles stretch on the slant board. The stretch was performed with an extended and flexed knee.	3 times, holding each stretch for 30 seconds
Strengthening	Calf raises Flexed knee calf raises Single-leg eccentric calf raises on a step	1 set of 15 repetitions

### Statistical analyses

The normality of data distribution was checked by the Shapiro-Wilk test. An independent t-test was used to compare the demographic information between the training group and the control group. A mixed factorial analysis of variance was used to determine any interaction between groups (training and control group) and time (pre- and post-intervention) factors. Then, pairwise comparisons were used to assess the differences between the pre-test and post-test in each group (training and control group) individually (within-group comparison). Bonferroni correction was applied for pairwise comparisons. Effect sizes (ES) using partial eta squared were calculated to increase the analysis power. ES were classified as small (0.01), moderate (0.06), and large (0.14) [30]. All statistical analyses performed in SPSS^®^ 26 software at the significance level were set to 95% (P < 0.05).

Based on the 95% confidence level in the current study, the minimal clinically important difference (MCID) was assessed using the distribution-based approach, while the minimal detectable change (MDC) was evaluated using the standard error of measurement (SEM) and the following equations [31]:

$$SEM=SD_{pre}\times \sqrt{1-rtest)}$$

$$MDC_{\%95}=1.96\times \sqrt{2}\times SEM$$

$$MCID_{RCI}=1.96\times SD_{pre}[\sqrt{\left(2\times (1-rtest)\right)}]$$

Where SD_pre_ is related to the training group, the retest is intraclass correlation coefficient (ICC) and MCID_RCI_ is the minimal clinically important difference of reliable change index. The Minimal Clinically Important Difference (MCID) is a key concept in clinical research, representing the smallest change in a treatment outcome that is considered significant. The distribution-based approach is one method used to determine the MCID, relying on statistical calculations rather than patient or clinician input. This approach is particularly valuable when direct patient feedback regarding their condition in relation to the intervention is unavailable [32, 33].

## Results

### Demographics

There were no significant differences between groups in any demographic variables (p> 0.05; Table 2).

**Table 2:** Comparison of the participants’ demographic information in the training and control group.

Variable	Mean± SD		P*
	Training Group	Control Group	
Age (years)	20.13 ± 2.26	20.40 ± 2.66	0.770
Height (cm)	161.66 ± 4.73	161.17 ± 4.15	0.767
Weight (kg)	58.79 ± 7.30	60.30 ± 11.78	0.676
BMI (kg/m^2^)	22.49 ± 2.66	23.21 ± 4.53	0.602
Sport history (years)	5.30 ± 1.88	5.61 ± 2.66	0.737

SD, Standard Deviation; BMI, Body Mass Index.

The p-value was derived from independent t-test.

### Range of motion

A mixed factorial analysis of variance revealed a significant interaction effect of group and time in ankle dorsiflexion ROM in a closed kinematic chain (P = 0.001; ES = 0.714; 95%CI = [-8.87%, -6.18%]). Results of pairwise comparisons demonstrated that ankle dorsiflexion ROM in a closed kinematic chain increased in the training group by approximately 7°, respectively from pre- to post-test (p < 0.05; Table 3).

**Table 3:** Effect of CCEP on ROM, proprioception, and balance.

Variable	Training Group (Mean ± SD)			Control Group (Mean ± SD)			Group*Time		
	Pre-test	Post-test	P	Pre-test	Post-test	P	F(1,23)	P	ES
**ROM (degree)**	32.57 ± 2.47	40.05 ± 4.33	0.001†	31.71 ± 2.25	31.71 ± 2.69	0.998	69.96	0.001‡	0.714
**Proprioception error (degree)**	6.77 ± 1.92	3.81 ± 2.33	0.001†	6.96 ± 2.90	8.57 ± 4.44	0.239	9.500	0.005‡	0.253
**Y-Balance Test (cm)**	84.96 ± 5.99	92.02 ± 5.20	0.001†	85.77 ± 9.15	86.14 ± 7.22	0.693	20.487	0.001‡	0.423

SD = Standard Deviation; ES = Effect Sizes. The symbol

† shows a significant difference between pre/post conditions and

‡ shows a significant interaction effect between the training and control group.

### Ankle proprioception

A mixed factorial analysis of variance revealed a significant interaction effect of group and time in proprioception (P = 0.005; ES = 0.253; 95%CI = [1.46%, 4.45%]). Results of pairwise comparisons demonstrated that repositioning error in the ankle decreased in the training group by approximately 3° from pre- to post-test (p < 0.05; Table 3).

### Dynamic balance

A mixed factorial analysis of variance revealed a significant interaction effect of group and time in dynamic balance (P = 0.001; ES = 0.423; 95%CI = [-9.57%, -4.56%]). Results of pairwise comparisons demonstrated that dynamic balance increased in the training group by 7 cm from pre- to post-test (p < 0.05; Table 3).

### Muscle activation

A mixed factorial analysis of variance revealed a significant interaction effect of group and time in tibialis anterior (P = 0.005; ES = 0.250; 95%CI = [-1.31%, 15.07%]), medial gastrocnemius (P = 0.048; ES = 0.132; 95%CI = [-1.49%, 7.08%]), and soleus (P = 0.008; ES = 0.223; 95%CI = 5.38%, 22.47%]) muscles activity in the descending phase of the overhead squat. Results of the pairwise comparisons in the descending phase of the overhead squat demonstrated that the muscle activity of the tibialis anterior, and soleus decreased in the training group by approximately 15 and 12%MVIC, respectively from pre- to post-test (p < 0.05; Table 4).

**Table 4:** Effect of CCEP on muscle activation index.

Variable		Training Group (Mean ± SD)			Control Group (Mean ± SD)			Group*Time		
		Pre-test	Post-test	P	Pre-test	Post-test	P	F(1,23)	P	ES
Tibialis anterior	descending	51.98 ± 9.96	37.39 ± 11.75	0.001†	49.36 ± 13.68	52.92 ± 23.05	0.543	9.322	0.005‡	0.250
	ascending	30.74 ± 13.24	22.10 ± 8.76	0.005†	29.34 ± 11.06	32.23 ± 12.67	0.251	10.448	0.003‡	0.272
Peroneus longus	descending	46.09 ± 23.88	42.59 ± 19.47	0.381	47.98 ± 21.84	49.90 ± 26.76	0.572	1.131	0.297	0.039
	ascending	32.16 ± 15.89	31.44 ± 19.82	0.773	34.34 ± 19.07	27.78 ± 15.21	0.208	1.106	0.302	0.038
Medial gastrocnemius	descending	17.67 ± 9.54	14.87 ± 8.42	0.184	16.49 ± 5.79	18.64 ± 6.44	0.123	4.273	0.048‡	0.132
	ascending	13.87 ± 10.12	12.14 ± 5.72	0.440	14.34 ± 6.20	15.91 ± 8.98	0.453	1.229	0.277	0.042
Soleus	descending	32.27 ± 13.22	20.34 ± 4.87	0.004†	28.90 ± 13.86	29.52 ± 18.87	0.824	8.021	0.008‡	0.223
	ascending	36.88 ± 12.26	22.69 ± 4.63	0.000†	37.73 ± 20.45	38.06 ± 20.21	0.801	19.891	0.001‡	0.415

SD = Standard Deviation; ES = Effect Sizes; all values are normalised to MVIC.

The symbol t21† shows a significant difference between pre/post conditions and

‡ shows a significant interaction effect between the training and control group.

A mixed factorial analysis of variance revealed a significant interaction effect of group and time in tibialis anterior (P = 0.003; ES = 0.272; 95%CI = [-4.01%, 6.57%]), and soleus (P = 0.001; ES = 0.415; 95%CI = [7.77%, 20.60%]) muscles activity in the ascending phase of the overhead squat. Results of the pairwise comparisons in the ascending phase of the overhead squat demonstrated that the muscle activity of the tibialis anterior, and soleus decreased in the training group by approximately nine and 14%MVIC, respectively from pre- to post-test (p < 0.05; Table 4).

It was observed that the mean pre/post difference in variables of the CKC ROM, proprioception, and balance test exceeded both MDC and MCID values. In the training group, the number of participants who had an average pre- and post- training improvement exceeding both the MDC and MCID were as follows: 15 for CKC ROM, 10 for proprioception, and 7 for balance. Conversely, in the control group, the respective numbers were 3 for CKC ROM, 4 for proprioception, and 0 for balance. This indicates that in addition to the changes being statistically significant, the value of changes obtained is also clinically significant (Table 5).

**Table 5:** The MDC and MCID values of the study variables.

Variable	SD_pre_ TG	Δ Score	SEM	MDC_%95_	MCID_RCI_
Closed kinetic chain (degree)	2.47	7.48	0.42	1.16	1.21
Proprioception (degree)	1.92	2.96	0.71	1.96	1.99
Y-Balance Test (cm)	5.99	7.06	2.33	6.44	6.46

SDpre TG = Standard Deviation Pre-Test Training Group; SEM = Standard Error of Measurement; MDC = Minimal Detectable Change; MCIDRCI = Minimal Clinically Important Difference of Reliable Change Index.

## Discussion

As an effort to provide scientific evidence for an effective training program, this randomized controlled trial investigated the efficacy of the CCEP including fascial and muscle release, talocrural joint mobilization, calf muscle stretching, and ankle muscles strengthening exercises in people with limited ankle dorsiflexion ROM. Our findings showed that this training increased ankle dorsiflexion ROM and improved balance and proprioception. The changes observed surpassed the MCID, indicating that the improvements were not only statistically significant but also meaningful from a clinical perspective. Also, MDC ensures that the observed changes are real and not due to measurement error or variability. Also, after eight weeks of intervention, the activity of the tibialis anterior and soleus muscles in the dynamic overhead squat test (descending and ascending phases) and the activity of the medial gastrocnemius in the descending phase decreased significantly. The effect size (ES) was found to be moderate to large, indicating that the training had a significant impact on the measured outcomes. No statistically significant change was observed in the activity of the peroneus longus muscle.

Limited ankle dorsiflexion ROM may be due to ligamentous restrictions, osteokinematics restrictions, or arthrokinematics restrictions and decreased extensibility of the plantar flexors [34]. According to our results, it seems that a combination of soft tissue release, joint mobilization, stretching, and strengthening exercises played an important role in improving these limitations.

Myofascial release is one of the main methods for restoring muscle tissue flexibility after injuries caused by sports or physical activities. With its long-duration mechanical force, the myofascial release technique intends to restore optimal length, decrease pain, and improve function [35]. The pressure associated with myofascial release causes the Golgi tendon organs to sense a change of tension in the muscle and respond to this high or prolonged tension by inducing relaxation of the muscle spindles [36]. Also, due to the step-by-step pressures during loading and unloading, the muscle’s mechanical (physiological) stiffness changes, which has many positive effects on improving the ROM [37]. Kim *et al*. (2017) stated that applying a stretch after myofascial release increases its effectiveness [29] because the viscoelastic components of the myofascial tissue are mechanically affected by static stretch. Static stretching of the neuro-fascial tissue at the end of the ROM is likely to reduce the excitability of the motoneuron through presynaptic and postsynaptic mechanisms [38, 39]. Finally, these cases lead to an increase in athletes’ tolerance to stretching and a decrease in the sensitivity of the stretch reflex, and as a result, provide the possibility of increasing the ROM.

Because mechanoreceptors and proprioceptors participate in joint position sense and movement through the change of muscle length and play a role in regulating muscle stiffness, it can be suggested that reducing muscle stiffness with the help of exercises has a direct effect on joint proprioception. By affecting non-contractile components, joint mobilization causes structural changes in the talocrural joint, and this, in turn, increases the inflow of sensory-motor information to the central nervous system [40]. Stretching exercises have also been reported to enhance proprioceptive input and improve proprioception by activating the muscle spindle and Golgi tendon organ [41]. An increase in proprioception is associated with an increase in athletes’ ability to balance control [42]. The intricate interplay between the sensory and musculoskeletal systems, both of which rely on information from proprioceptive receptors, leads to the development of balance [43]. It seems that eight weeks of CCEP including soft tissue mobilization, joint mobilization, stretching, and strengthening can prevent ankle sprain injury by improving limited ankle dorsiflexion.

A significant decrease in medial gastrocnemius, soleus, and tibialis anterior activity was observed in the training group after the intervention. The calf muscle is one of the primary muscles that eccentrically resist ankle dorsiflexion [44]. Studies show that people with limited ankle dorsiflexion have calf muscle overactivity during the overhead squat task [45, 46]. This issue can lead to the shortening of the connective tissues of the gastrosoleus, Achilles tendon, and plantar fascia. Also, increasing the gastrocnemius activation may lead to a greater internal ankle plantar-flexion moment and increased posterior ankle stiffness [47].

Limited ankle dorsiflexion restricts the ability to downwardly move the body’s center of mass during tasks that involve weight bearing. As a result, adaptations such as increased rear-foot pronation and tibial internal rotation, ankle sprain, knee injury, and changes in lower limb muscle activation may occur [48, 49]. Padua *et al*. (2012) stated that reduced gastrocnemius and tibialis anterior activation during squatting conditions minimized the internal plantar-flexion moment and stiffness of the ankle, thus allowing for less restricted dorsiflexion and minimizing compensatory pronation [47]. Previous researchers demonstrated that increased coactivation of agonist and antagonist musculature enhanced overall joint stiffness [50, 51]. Increased tibialis anterior and calf muscle activation demonstrate greater overall coactivation in lower leg musculature, which can lead to limitation of dorsiflexion. Therefore, it seems that the CCEP used in this study, by reducing the activity of the gastrosoleus and tibialis anterior muscles, might have led to a reduction in joint stiffness and improvement in ankle dorsiflexion ROM.

The findings of our study align with and expand upon previous research in this area. For instance, Lee et al. (2021) examined the combined effects of gastrocnemius stretching and tibialis anterior resistance exercises on ankle kinematics and muscle activity in subjects with limited ankle dorsiflexion ROM. They found that adding tibialis anterior resistance exercise with gastrocnemius stretching significantly increased both the active ROM of ankle dorsiflexion and tibialis anterior muscle activity compared to gastrocnemius stretching alone [52]. Our study indicates that the CCEP not only enhanced ankle dorsiflexion ROM but also improved overall ankle function and stability, suggesting that a multifaceted exercise program may provide additional benefits over isolated stretching and resistance exercises. Furthermore, our results are consistent with the findings of Lee et al. (2021), who reported that the Graston technique was particularly effective in improving muscle balance and kinematics in individuals with limited ankle dorsiflexion [53]. Similarly, we observed that integrating manual techniques into our corrective exercise program significantly enhanced muscle function and joint mobility. However, our approach differed by incorporating a variety of manual and corrective exercises, which may explain the broader improvements in ankle function observed in our participants. Overall, while our study shares similarities with previous research regarding the positive impact of specific techniques on ankle dorsiflexion, it also emphasizes the added value of a comprehensive corrective exercise program in addressing multiple aspects of ankle function in female athletes.

This study is not without limitations. First, the sample size was small and could render our study underpowered. Second, our participants were only women and it could compromise the generalizability of our findings. Third, we did not assess the lower extremity muscle strength of our participants. It would be beneficial to examine this variable in future studies. Last, our results are limited to an overhead squatting task because we neither observed muscle activation during other functional tasks, such as jump landings or cutting maneuvers. Future authors should identify whether findings carry over when individuals perform more demanding tasks, such as cutting and jump landings.

## Supporting information

S1 Checklist The CONSORT (Consolidated Standards of Reporting Trials) guidelines are a set of recommendations designed to improve the reporting of randomized controlled trials (RCTs). (DOC)

S1 Protocol Protocol for ethics committee-English version. (DOCX)

S2 Protocol Protocol for ethics committee-Persian version. (DOCX)
