# Supplementary material for: Comprehensive corrective exercise program improves ankle function in female athletes with limited weight-bearing ankle dorsiflexion: A randomized controlled trial
Source: PLoS One. 2024 Oct 31;19(10):e0312152. doi: 10.1371/journal.pone.0312152 (PMC11527180; doi:10.1371/journal.pone.0312152)
Supplement: S1 Protocol — (DOCX) [file pone.0312152.s002.docx]

**Study protocol**

| Title | The effect of eight weeks of corrective intervention on lower extremity muscle electrical activity in women with limited ankle dorsiflexion |
| --- | --- |
| Student profile | Tahereh Sohrabi; Department of Exercise Rehabilitation, Faculty of Sport Sciences, Bu-Ali Sina University, Hamedan, Iran. |
| Profile of professors | Farzaneh Saki; Department of Exercise Rehabilitation, Faculty of Sport Sciences, Bu-Ali Sina University, Hamedan, Iran.  Behdad Tahayori; Department of Physical Therapy, University of Saint Augustine for Health Sciences, Miami, FL, USA |
| place | Bu-Ali Sina University |
| Financial institution | NA |

**Introduction**

Limited ankle dorsiflexion is a significant risk factor for various issues, including ankle sprains, knee injuries, gait abnormalities, balance deficits, and disturbances in dynamic activities. A reduced range of motion in ankle dorsiflexion has been identified as a potential cause of excessive knee valgus. Some researchers suggest that decreased ankle dorsiflexion can lead to increased subtalar pronation through abduction and internal rotation of the tibia, resulting in compensatory movements in the ankle and knee, ultimately causing knee valgus. Moreover, limited ankle dorsiflexion range of motion is a critical risk factor for ankle sprains, as it can disrupt the neuromuscular control of the muscles surrounding the ankle. Muscle imbalances in the leg, such as stiffness in the soleus, external gastrocnemius, and peroneal muscles, may contribute to the abduction and external rotation of the tibia, facilitating increased inward knee movement and knee valgus dynamics. Additionally, weakness in the internal gastrocnemius, tibialis anterior, and tibialis posterior muscles may reduce the ability to control foot pronation, leading to an increase in knee valgus dynamics. An increased valgus angle of the knee during dynamic movements, such as squats, is a risk factor for knee injuries and reduces the mechanical efficiency of the movement. There is a positive and significant relationship between ankle dorsiflexion range of motion and knee and ankle movement in the sagittal plane during squats. The connection between limited ankle dorsiflexion and various foot and ankle problems can be attributed to ligament, osteokinematic, and arthrokinematic limitations, as well as reduced extensibility of the ankle plantar flexors (1).

Preventing injuries, particularly in lower limb joints like the ankle, has always been a key focus for researchers. One of the primary exercises for preventing and treating ankle dorsiflexion limitations is stretching the muscles at the back of the leg (2). Several studies have highlighted the positive effects of stretching the gastrosoleus complex on the ankle joint's range of motion (3,4). However, a closer examination reveals that many studies overlook the capsular limitations affecting the range of motion and the significant role of the soleus muscle in activities involving knee flexion, which is crucial in many sports (5). Since talocrural dorsiflexion limitations may result from muscle stiffness and insufficient lateral movements, addressing these issues requires not only muscle stretching but also talocrural mobilization (6). Additionally, it's important to consider the benefits of fascia and muscle release techniques, such as using a foam roller and Graston, to enhance muscle function and joint range of motion. Research on the impact of ankle dorsiflexion limitations on the surrounding muscles is limited. Most existing studies focus on non-athletes or individuals without natural dorsiflexion limitations, where researchers manipulated the range of motion through interventions. Therefore, it is essential to investigate the activation patterns and levels of ankle muscles in athletes with natural dorsiflexion limitations, as this is a risk factor for ankle sprains. A review of previous literature suggests that significant improvements in the talocrural range of motion can be achieved through interventions targeting stiffness and posterior glide insufficiency (7). The Graston technique, specifically used to diagnose and treat soft tissue dysfunction, is believed to enhance musculoskeletal function and facilitate pain-free movement (8). Given the conflicting study results and the importance of injury prevention, it is crucial to develop the most effective corrective program (9). Choosing the wrong program can fail to address abnormalities and pathologies, wasting both time and money.

Thus, the key question is whether eight weeks of corrective interventions can improve the dorsiflexion range of motion, ankle proprioception, dynamic balance, and electromyographic activity of selected ankle muscles during the overhead squat test in athletes with limited ankle dorsiflexion.

**Study Aims**

The research aims are divided into two categories: primary and secondary aims.

1. Primary Aim

To determine the effect of an eight-week corrective intervention in women with limited ankle dorsiflexion.

2. Secondary Aims

- To assess the effect of an eight-week corrective intervention on the ankle range of motion in women with limited ankle dorsiflexion.
- To assess the effect of an eight-week corrective intervention on ankle proprioception in women with limited ankle dorsiflexion.
- To assess the effect of an eight-week corrective intervention on dynamic balance in women with limited ankle dorsiflexion.
- To assess the effect of an eight-week corrective intervention on the muscle activity of the anterior tibialis, peroneus longus, medial gastrocnemius, and soleus muscles during the overhead squat test in women with limited ankle dorsiflexion.

**Study Hypotheses**

- Eight weeks of corrective intervention will improve ankle range of motion in women with limited ankle dorsiflexion.
- Eight weeks of corrective intervention will improve proprioception in women with limited ankle dorsiflexion.
- Eight weeks of corrective intervention will improve dynamic balance in women with limited ankle dorsiflexion.
- Eight weeks of corrective intervention will improve the muscle activity of the tibialis anterior, peroneus longus, medial gastrocnemius, and soleus muscles during the overhead squat test in women with limited ankle dorsiflexion.

**Study Variables**

- - Independent Variable

The independent variable in this study is the corrective exercises, which include fascia and posterior leg muscle release, gastrosoleus muscle group stretching, talocrural joint mobilization, and strengthening exercises.

- - Dependent Variables

The dependent variables are the electromyography activity levels of selected lower limb muscles, ankle range of motion, proprioception, and dynamic balance.

**Materials and Methods**

**Study Design**

Randomized controlled trial.

**Population**

Inclusion Criteria:

- Female athletes
- Age range: 15 to 25 years
- Athletes with limited ankle dorsiflexion (≤ 34°) during the weight-bearing lunge test
- Training at least three sessions per week (60 minutes per session)
- Body mass index between 20 and 25 kg/m²

Exclusion Criteria:

- - Presence of pain
  - History of lower limb surgery
  - Lower limb injury within the last six months
  - Contraindications to soft tissue mobilization with instruments, including a history of arteriosclerosis, thrombosis, embolism, severe varicose veins, acute phlebitis, cellulitis, synovitis, abscess, skin infections, cancers, and acute inflammatory conditions

**Sampling Method and Sample Size**

The sample size was calculated using G-Power software with a test power of 0.85, an average effect size of 0.5, and a significance level of 0.05. Considering potential dropouts, 30 participants will be selected as the statistical sample. 15 participants will be in each of the intervention and control groups. Participants will be purposefully selected from athletes with limited ankle dorsiflexion. After identifying eligible athletes based on the inclusion and exclusion criteria, they will be invited to participate in the study. Those who agree will be asked to attend the corrective movements laboratory at the Faculty of Physical Education, Bu-Ali Sina University. The 30 selected participants will then be randomly assigned to either the intervention or control groups, with 15 participants in each.

**Study Process**

Written informed consent to participate will be obtained from all participants' parents or legal guardians before their involvement in the study. Participation in the study will be voluntary, and participants can withdraw at any time without affecting their access to usual care, diagnosis, and treatment services. There will be no costs associated with participation, and all collected information will remain confidential. After registration, athletes will be divided into two groups: the exercise group, which received the corrective interventions for eight weeks, and the control group, which continued their regular team training. Participants will be randomly assigned to groups using Random Number Generator software, and allocation concealment will be maintained using sequentially numbered opaque sealed envelopes.

**Research Measurement Process**

1. The examiner interviews to complete the information collection form.

2. Participants complete the consent form.

3. Measure height and weight.

4. Assess the range of motion for dorsiflexion.

5. Evaluate ankle proprioception.

6. Measure dynamic balance.

7. Assess the Maximum Voluntary Isometric Contraction (MVIC) of the studied muscles.

8. Record the activity of selected muscles during the overhead squat movement.

9. Do the training protocol over eight weeks, with three sessions per week, each lasting 30 minutes.

10. Conduct post-testing and re-measure all study variables (steps 4 to 8).

**Intervention**

The corrective intervention protocol includes warm-up, soft tissue mobilization, joint mobilization, stretching, and strengthening. The protocol will be performed 30 minutes a day for eight weeks (three sessions per week; 24 sessions). At first, participants will perform a 15-minute warm-up protocol focusing on the lower extremity muscles. Then, the participants lay prone on an examination table with the hip in full extension, the knee in partial flexion, and the ankle in plantar flexion. The gastrocnemius muscle, Achilles tendon, and plantar fascia will smear with Graston Technique (GT) emollient. At first, a sweeping stroke will used to scan the adhesions for one minute with the GT instrument. Then, four minutes will used to focus on adhesions for the remaining. For the first six sessions, GT instruments will be used at a 30° to 45° angle while moderate pressure is maintained, and sweeping, fanning, and scooping strokes will be used in all directions. From the seventh to twelfth sessions, the GT instruments will be used at about 60° angle and with more pressure. After the twelfth session, a foam roller will used for 30 seconds to release the gastrosoleus muscles as self-mobilization. Following the soft tissue mobilization participants will be asked to perform stretches of the gastrosoleus muscles and mobilize the talocrural joint in a standing position with the use of a strap. To do this movement, participants position the knee of the leg being trained approximately 12-14 cm ahead of the non-training leg. A strap secured to a stable object will be positioned around the front part of the ankle joint. Participants will then be guided to gently move the knee of the trailing leg forward into dorsiflexion, ensuring the movement is pain-free. Maintaining contact between the heel of the trailing leg and the ground is important throughout the exercise. Finally, the participant performs strength exercises as part of the corrective interventions. We will customize the exercises for each athlete, based on the individual's needs and progress throughout the intervention. The initial phase will focus on familiarizing participants with the correct form and execution of the exercises. As participants demonstrate proficiency and improved strength and flexibility, the intensity and complexity of the exercises will progressively be increased. This includes variations in the number of sets, repetitions, and resistance used, to continually challenge the muscles and prevent plateauing. The progression will be carefully monitored and adjusted based on individual responses to ensure safety and optimal benefit from the program.

**Statistical Method**

Descriptive and inferential statistical methods will be used to analyze the collected data. The Shapiro-Wilk test will be employed to determine the normality of the data. To investigate the interactive effect of time (with two levels: before and after the intervention) on the group (exercise and control) in the values of the variables, a mixed analysis of variance with repeated measures will be conducted. Data analysis will be performed at a 95% significance level, with an alpha level of 0.05 or less, using SPSS version 26 software.

1. Young R, Nix S, Wholohan A, Bradhurst R, Reed L. Interventions for increasing ankle joint dorsiflexion: a systematic review and meta-analysis. Journal of foot and ankle research. 2013;6(1):1-10.

2. Dinh N, Freeman H, Granger J, Wong S, Johanson M. Calf stretching in non-weight bearing versus weight bearing. International journal of sports medicine. 2011;32(03):205-10.

3. Bryan Dixon J. Gastrocnemius vs. soleus strain: how to differentiate and deal with calf muscle injuries. Current reviews in musculoskeletal medicine. 2009;2(2):74-7.

4. Macklin K, Healy A, Chockalingam N. The effect of calf muscle stretching exercises on ankle joint dorsiflexion and dynamic foot pressures, force and related temporal parameters. The Foot. 2012;22(1):10-7.

5. Howe L. Restricted ankle dorsiflexion: Methods to assess and improve joint function. Prof J Strength Cond. 2015;37:7-15.

6. Kang M-H, Lee D-K, Kim S-Y, Kim J-S, Oh J-S. The influence of gastrocnemius stretching combined with joint mobilization on weight-bearing ankle dorsiflexion passive range of motion. Journal of Physical Therapy Science. 2015;27(5):1317-8.

7. Kang M-H, Oh J-S, Kwon O-Y, Weon J-H, An D-H, Yoo W-G. Immediate combined effect of gastrocnemius stretching and sustained talocrural joint mobilization in individuals with limited ankle dorsiflexion: A randomized controlled trial. Manual Therapy. 2015;20(6):827-34.

8. Laudner K, Compton BD, McLoda TA, Walters CM. Acute effects of instrument assisted soft tissue mobilization for improving posterior shoulder range of motion in collegiate baseball players. International journal of sports physical therapy. 2014;9(1):1.

9. Carey-Loghmani M, Schrader J, Hammer W. Clinical foundations for graston technique® adapted from: graston technique® m1 instruction manual. 2014.
