# Supplementary material for: Comprehensive corrective exercise program improves ankle function in female athletes with limited weight-bearing ankle dorsiflexion: A randomized controlled trial
Source: PLoS One. 2024 Oct 31;19(10):e0312152. doi: 10.1371/journal.pone.0312152 (PMC11527180; doi:10.1371/journal.pone.0312152)
Supplement: S2 Protocol — (DOCX) [file pone.0312152.s003.docx]

## **پروتکل مطالعاتی**

| عنوان | تأثیر هشت هفته مداخلة اصلاحی بر فعالیت الکتریکی عضلات اندام تحتانی در زنان دارای محدودیت دورسی­فلکشن مچ پا |
| --- | --- |
| مشخصات دانشجو | طاهره سهرابی، دانشجو کارشناسی ارشد، گروه توانبخشی ورزشی، دانشکده علوم ورزشی، دانشگاه بوعلی سینا، همدان، ایران. |
| مشخصات اساتید | فرزانه ساکی، گروه توانبخشی ورزشی، دانشکده علوم ورزشی، دانشگاه بوعلی سینا، همدان، ایران. بهداد تحیری، گروه فیزیوتراپی، دانشگاه سنت آگوستین برای علوم بهداشت، میامی، فیلادلفیا، ایالات متحده آمریکا. |
| مکان | دانشگاه بوعلی سینا همدان |
| نهاد مالی | ندارد. |

## **بیان مسئله**

محدودیت حرکتی دورسی­فلکشن مچ پا یک عامل خطر برای پیچ‌خوردگی مچ پا، افزایش آسیب‌های زانو، ناهنجاری‌های راه رفتن، نقص تعادل و اختلال در فعالیت‌های پویا است. محدودیت دامنة حرکتی دورسی­فلکشن به‌عنوان یکی از عوامل احتمالی در ایجاد والگوس بیش از حد زانو گزارش شده است چرا که برخی محققان معتقدند کاهش دامنة حرکتی دورسی­فلکشن مچ پا می‌تواند به وسیله­ی ابداکشن و اینترنال روتیشن تیبیا، افزایش پرونیشن ساب تالار موجب حرکات جبرانی در مچ پا و زانو و ایجاد والگوس زانو شود. از طرفی یکی از مهم‌ترین عوامل خطرساز بروز آسیب اسپرین مچ پا محدودیت دامنة حرکتی دورسی­فلکشن می‌باشد که می‌تواند کنترل عصبی - عضلانی عضلات اطراف مچ پا را دچار اختلال کند. عدم تعادل عضلانی در ساق پا شامل سفتی عضلات سولئوس، گاستروکنمیوس خارجی و عضلات پرونئال ممکن است به ابداکشن و اکسترنال روتیشن تیبیا و سهولت در افزایش حرکت زانو به سمت داخل و راستای والگوس داینامیک زانو کمک کند. همچنین ضعف گاستروکنمیوس داخلی، تیبیالیس­قدامی و تیبیالیس­خلفی ممکن است توانایی کنترل حرکات پرونیشن پا را کاهش دهد و به افزایش والگوس داینامیک زانو منجر شود. افزایش زاویه والگوس زانو در خلال حرکات پویا نظیر اسکات یکی از عوامل خطرآفرین آسیب زانو و کاهش بازده مکانیکی حرکت است. بین دامنة حرکتی دورسی­فلکشن مچ پا با حرکت زانو و مچ در صفحة ساجیتال حین اسکات ارتباط مثبت و معنی‌داری وجود دارد. آنچه به‌خوبی قابل استناد است ارتباط بین محدودیت دامنة حرکتی دورسی­فلکشن مچ پا با بسیاری از مشکلات پا و مچ پا است (1) که این محدودیت ممکن است ناشی از محدودیت‌های لیگامانی، استئوکینماتیک، آرتروکینماتیک و کاهش انبساط‌پذیری پلانتار فلکسورهای مچ پا باشد.

پیشگیری از آسیب باتوجه‌به شیوع قابل‌توجه آن در مفاصل اندام تحتانی به‌ویژه مفصل مچ، همواره مورد توجه محققین مختلف بوده است. یکی از تمرین‌های اصلی حوزه پیشگیری و درمان محدودیت دورسی­فلکشن، تمرینات کششی عضلات پشت ساق پا می‌باشد (2). دراین رابطه، چندین مطالعه اثرات مثبت برنامه کششی مجموعه گاستروسولئوس بر دامنة حرکتی مفصل مچ پا را گزارش نموده­اند (3, 4). بااین‌وجود، با نگاهی دقیق­تر می­توان دریافت که در اکثر تحقیقات صورت‌گرفته، محدودیت کپسولار مرتبط با محدودیت دامنة حرکتی و نقش پررنگ عضله سولئوس در فعالیت­های همراه با فلکشن زانو که اکثر فعالیت­های مهم ورزشی را شامل می­شود نادیده گرفته شده است (5). ازآنجایی‌که محدودیت حرکتی دورسی­فلکشن تالوکرال ممکن است ناشی از سفتی عضلات و همچنین حرکات جانبی ناکافی تالوکرال باشد، برای تصحیح حرکات جبرانی در طی حرکات پویا و رفع محدودیت، علاوه بر کشش عضلات باید موبیلیزیشن تالوکرال را نیز مدنظر داشت (6). علاوه‌برآن توجه به تأثیر استفاده از روش­های ریلیز فاشیا و عضله با استفاده از فوم رولر و گراستون در بهبود عملکرد عضله و در نهایت دامنة حرکتی مفاصل نیز حائز اهمیت است. تاکنون تحقیقات اندکی در خصوص تأثیر محدودیت دامنة حرکتی دورسی­فلکشن مچ پا بر فعالیت عضلات اطراف مچ پا انجام شده است. اندک تحقیقاتی نیز که دراین‌خصوص انجام شده است، بیشتر بر روی افرادی انجام شده است که یا ورزشکار نبوده‌اند و یا خود به طور طبیعی دارای محدودیت حرکتی دورسی­فلکشن نبوده‌اند و محقق در آن تحقیقات با اعمال یک مداخله، میزان دامنة حرکتی را دست‌کاری کرده است؛ بنابراین بررسی الگو و میزان فعال‌سازی عضلات مچ پا در افراد ورزشکاری که خود به طور طبیعی دارای محدودیت حرکتی دورسی­فلکشن هستند، به‌عنوان یکی از عوامل خطرزای آسیب اسپرین مچ پا، ضروری به نظر می‌رسد. باتوجه‌به بررسی ادبیات قبلی، به نظر می­رسد افزایش معنی­داری در دامنة حرکتی تالوکرال بعد از اعمال مداخله­هایی برای رفع سفتی و ناکافی بودن گلاید خلفی تالوکرال مشاهده شده است (7). ریلیز فاشیا و عضله به‌وسیلة گراستون به طور ویژه برای تشخیص و درمان اختلال عملکرد بافت نرم به کار می­رود (8). اعتقاد بر این است که تکنیک گراستون عملکرد اسکلتی - عضلانی و حرکت بدون درد را بهبود می­بخشد (9). به جهت ضدونقیض بودن نتایج مطالعات و اهمیت موضوع پیشگیری از آسیب لازم است تا مؤثرترین و بهترین برنامه اصلاحی فراهم شود، چرا که عدم انتخاب برنامه مناسب باعث عدم بهبودی در وضعیت ناهنجاری­ها و بروز پاتولوژی در سیستم و همچنین صرف و تلف‌شدن هزینه و وقت فرد خواهد گردید.

بنابراین سوال حقیق حاضر این است که آیا هشت هفته مداخلات اصلاحی (شامل: ریلیز فاشیا و عضلات خلف ساق پا، تمرینات کششی گروه عضلانی گاستروسولئوس، موبیلیزیشن مفصل تالوکورال و تمرینات تقویتی) بر بهبود دامنة حرکتی دورسی­فلکشن، حس عمقی مچ پا، تعادل پویا و فعالیت الکترومایوگرافی عضلات منتخب مچ پا در تست اسکات بالای سر در ورزشکاران دارای محدودیت حرکتی دورسی­فلکشن تأثیر دارد؟

## **اهداف پژوهش**

اهداف تحقیق در دو مبحث هدف کلی و اهداف اختصاصی ارائه می‌شود.

### هدف کلی

تعیین تأثیر هشت هفته مداخلة اصلاحی بر دامنة حرکتی مچ پا، حس عمقی، تعادل پویا و فعالیت الکتریکی عضلات اندام تحتانی در زنان دارای محدودیت دورسی­فلکشن مچ پا.

### اهداف جزئی

- تعیین تأثیر هشت هفته مداخله اصلاحی بر دامنة حرکتی زنجیره زنان دارای محدودیت حرکتی دورسی­فلکشن.
- تعیین تأثیر هشت هفته مداخله اصلاحی بر حس عمقی زنان دارای محدودیت حرکتی دورسی­فلکشن.
- تعیین تأثیر هشت هفته مداخله اصلاحی بر تعادل پویا زنان دارای محدودیت حرکتی دورسی­فلکشن.
- تعیین تأثیر هشت هفته مداخله اصلاحی بر فعالیت الکتریکی عضلات تیبیالیس قدامی، پرونئوس لانگوس، گاستروکنمیوس داخلی و سولئوس زنان دارای محدودیت حرکتی دورسی­فلکشن در طی تست اسکات بالای سر.

## **فرضیه­های پژوهش**

- هشت هفته مداخله اصلاحی بر بهبود دامنة حرکتی مچ پا زنان دارای محدودیت حرکتی دورسی­فلکشن تأثیر دارد.
- هشت هفته مداخله اصلاحی بر بهبود حس عمقی زنان دارای محدودیت حرکتی دورسی­فلکشن تأثیر دارد.
- هشت هفته مداخله اصلاحی بر بهبود تعادل پویا زنان دارای محدودیت حرکتی دورسی­فلکشن تأثیر دارد.
- هشت هفته مداخله اصلاحی بر فعالیت الکتریکی عضلات تیبیالیس قدامی، پرونئوس لانگوس، گاستروکنمیوس داخلی و سولئوس زنان دارای محدودیت حرکتی دورسی­فلکشن طی تست اسکات بالای سر تأثیر دارد.

**متغیرهای تحقیق**

- متغیر مستقل

متغیر مستقل در این تحقیق تمرینات اصلاحی (شامل: ریلیز فاشیا و عضلات خلف ساق پا، تمرینات کششی گروه عضلانی گاستروسولئوس، موبیلیزیشن مفصل تالوکرال و تمرینات تقویتی) است.

- متغیر وابسته

میزان فعالیت الکترومیوگرافی عضلات منتخب اندام تحتانی (تیبیالیس قدامی، پرونئوس لانگوس، گاستروکنمیوس داخلی و سولئوس)، دامنة حرکتی، حس عمقی و تعادل پویا مفصل مچ پا متغیرهای وابسته این تحقیق هستند.

**مواد و روش**

**طراحی مطالعه**

کارآزمایی کنترل تصادفی

**جمعیت**

معیارهای ورود:

- دختران ورزشکار رشته دو و میدانی
- رنج سنی بین 15 تا 25 سال
- ورزشکاران با دورسی فلکشن محدود مچ پا در طول تست لانژ تحمل وزن (≤ 34◦)
- تمرین حداقل سه جلسه در هفته (هر جلسه 60 دقیقه)
- شاخص توده بدنی 20 تا 25 کیلوگرم بر متر مربع

معیارهای خروج:

- درد
- سابقه عمل جراحی اندام تحتانی
- آسیب اندام تحتانی در شش ماه گذشته
- موارد منع حرکت بافت نرم با کمک ابزار شامل سابقه تصلب شرایین، ترومبوز، آمبولی، وریدهای واریسی شدید، فلبیت حاد، سلولیت، سینوویت، آبسه، عفونت های پوستی، سرطان ها و شرایط التهابی حاد.

## **روش نمونه‌گیری و حجم نمونه**

تعداد نمونه­های تحقیق با استفاده از نرم‌افزار G-Power (توان آزمون = 85/0، اندازة اثر متوسط = 5/0 و سطح معناداری = 05/0) 12 محاسبه شد که با درنظرگرفتن احتمال ریزش آزمودنی­ها تعداد 30 نفر به‌عنوان نمونه آماری انتخاب می شوند؛ بنابراین تعداد افراد حاضر در هر یک از گروه­های تمرین و کنترل 15 نفر در نظر خواهد شد. پس از مشخص‌شدن حجم نمونه، انتخاب نمونه­ها به‌صورت هدفمند از بین ورزشکاران دارای محدودیت حرکتی دورسی فلکشن صورت خواهد پذیرفت. نحوة گزینش نمونه­ها به این شکل میباشد که پس از شناسایی ورزشکاران دارای محدودیت دورسی فلکشن از میان جامعة آماری و بر اساس معیارهای ورود و خروج تحقیق، از آنها دعوت به عمل می آید تا در صورت تمایل به همکاری در تحقیق حاضر، در زمان مشخص در آزمایشگاه حرکات اصلاحی دانشکدة تربیت‌بدنی دانشگاه بوعلی سینا حضور یابند. پس از حضور افراد منتخب تعداد 30 نفر از آنها به‌صورت هدفمند در دو گروه 15 نفری (یک گروه تمرین و یک گروه کنترل) قرار داده میشوند.

**روند پژوهش**

به منظور حصول اطمینان از رضایت افراد برای شرکت در این پژوهش، فرم رضایت نامه ی کتبی در اختیار آنان قرار خواهد گرفت و تمام خطرات احتمالی آزمون برای آزمودنی شرح داده خواهد شد. برای افراد زیر سن قانونی رضایت کتبی از والدین آنها کسب خواهد شد. افراد شرکت کننده در آزمون برای شرکت یا عدم شرکت در آزمون آزاد هستند و پس از اعلام موافقت خود نیز، هرزمانی که بخواهند می توانند از آزمون خارج شوند. خروج از پزوهش باعث محرومیت آزمودنی ها از دریافت خدمات مراقبتی ، تشخیصی و درمانی معمول نخواهد شد و مستلزم پرداخت جریمه نخواهند بود. هیچ گونه هزینه ای جهت شرکت در این تحقیق بر عهده آزمودنی نیست و تمامی اطلاعاتی که جهت شرکت در آزمون از آزمودنی گرفته خواهد شد محرمانه خواهد ماند. پس از ثبت نام، ورزشکاران در دو گروه تجربی و کنترل طبقه بندی می شوند. گروه تمرینی به مدت هشت هفته مداخلات اصلاحی مورد نظر را دریافت میکنند درحالیکه گروه کنترل در این هشت هفته به تمرینات روزمره تیمی خود خواهند پرداخت. با استفاده از نرم افزار Random Number Generator، شرکت کنندگان به طور تصادفی در یکی از گروه ها قرار خواهند گرفت. پنهان سازی تخصیص با تکنیک پاکت های مهر و موم شده مات با شماره ترتیبی انجام می شود.

## **روند اندازه­گیری­های پژوهش**

1. تکمیل فرم جمع­آوری اطلاعات توسط آزمونگر از طریق مصاحبه.

2. تکمیل فرم رضایت­نامه توسط نمونه­های انتخاب شده.

3. اندازه­گیری قد، وزن.

4. اندازه­گیری دامنة حرکتی دورسی­فلکشن.

5. اندازه­گیری حس عمقی مچ پا.

6. اندازه گیری تعادل پویا.

7. اندازه‌گیری MVIC عضلات مورد مطالعه.

8. ثبت فعالیت عضلات منتخب حین حرکت اسکات بالای سر.

9. اجرای پروتکل تمرینی به مدت هشت هفته سه جلسه­ای که طول هر جلسه 30 دقیقه بود.

10. انجام پس­آزمون و اندازه‌گیری مجدد تمام متغیرهای مطالعه (مرحله 4 تا 8).

## **مداخله اصلاحی**

24 جلسه تمرین در یک دوره هشت هفته­ای انجام میشود؛ بنابراین گروه تجربی پروتکل موردنظر را سه بار در هفته اجرا خواهد کرد درحالی‌که گروه کنترل تمرینی نخواهند داشت. پروتکل جامع پژوهش حاضر شامل: ریلیز فاشیا و عضلات خلف ساق پا با استفاده از گراستون و فوم رولر، موبیلیزیشن مفصل تالوکرال با استفاده از بند غیر انعطاف، تمرینات کششی گروه عضلانی گاستروسولئوس و تمرینات تقویتی است.

در هر جلسه، به آزمودنی‌ها آموزش داده میشود تا به مدت 15 دقیقه پروتکل گرم‌کردن را با تمرکز بر عضلات اندام تحتانی انجام دهند. سپس شرکت‌کنندگان به‌طوریکه ران در اکستنشن کامل، زانو در فلکشن جزئی و مچ پا در پلنتار فلکشن قرار داشته باشد، به حالت پرون روی تخت دراز می‌کشند. سپس عضله گاستروکنیموس، تاندون آشیل و فاشیای کف پا به روغن ماساژ آغشته میشود. در ابتدا با ابزار گراستون برای اسکن چسبندگی‌های به مدت 1 دقیقه از حرکت رفت و برگشتی استفاده میشود. سپس، به مدت 4 دقیقه به ریلیز چسبندگی‌ها اختصاص داده خواهد شد. برای شش جلسه اول، از ابزارها با زاویه 30 تا 45 درجه درحالی‌که فشار متوسط در طی حرکت‌های اسکوپینگ، رفت و برگشتی و بادبزنی ​​اعمال می‌شود، استفاده خواهد شد. در طی جلسات هفت تا 12، ابزار با زاویه 60 درجه و با فشار بیشتری مورد استفاده قرار خواهد گرفت. بعد از جلسه دوازدهم از فوم رولر به مدت 30 ثانیه برای رهاسازی گروه عضلانی گاستروسولئوس استفاده میشود. سپس از شرکت‌کنندگان خواسته میشود که کشش ساق پا را با استفاده از سطح شیب‌دار انجام دهند و هر کشش را به مدت 30 ثانیه نگه دارند. کشش سه بار با زانوی صاف و سه بار با زانوی خمیده انجام خواهد شد. در مرحله بعد فرد کشش گاستروسولئوس به همراه موبیلیزیشن مفصل تالوکرال را در حالت ایستاده انجام میشود. این کار در پنج نوبت و با 30 ثانیه کشش و 30 ثانیه استراحت انجام خواهد شد. در نهایت، شرکت‌کننده یک ست، 15 تکرار تمرینات رفتن روی پنجه با زانو صاف، روی پنجه رفتن با زانوی خمیده و رفتن روی پنجه پا به صورت تک پا و با استفاده از یک پله را انجام می­دهند.

## **روش آماری**

برای تجزیه‌وتحلیل اطلاعات جمع‌آوری‌شده از روش­های آماری توصیفی و استنباطی استفاده می­شود. جهت تشخیص نرمال بودن داده­ها، آزمون شاپیروویلک و همچنین برای بررسی اثر تعاملی زمان (دارای دو سطح قبل از مداخله و بعد از مداخله) بر گروه (تمرین و کنترل)، در مقادیر متغیرها آنالیز واریانس ترکیبی با اندازه­های تکراری مورد استفاده قرار می­گیرد. تجزیه‌وتحلیل اطلاعات در سطح معنی‌داری 95% و میزان آلفای کوچک‌تر یا مساوی 05/0 و با استفاده از نرم‌افزار SPSS نسخه 26 انجام خواهد شد.

1. Young R, Nix S, Wholohan A, Bradhurst R, Reed L. Interventions for increasing ankle joint dorsiflexion: a systematic review and meta-analysis. Journal of foot and ankle research. 2013;6(1):1-10.

2. Dinh N, Freeman H, Granger J, Wong S, Johanson M. Calf stretching in non-weight bearing versus weight bearing. International journal of sports medicine. 2011;32(03):205-10.

3. Bryan Dixon J. Gastrocnemius vs. soleus strain: how to differentiate and deal with calf muscle injuries. Current reviews in musculoskeletal medicine. 2009;2(2):74-7.

4. Macklin K, Healy A, Chockalingam N. The effect of calf muscle stretching exercises on ankle joint dorsiflexion and dynamic foot pressures, force and related temporal parameters. The Foot. 2012;22(1):10-7.

5. Howe L. Restricted ankle dorsiflexion: Methods to assess and improve joint function. Prof J Strength Cond. 2015;37:7-15.

6. Kang M-H, Lee D-K, Kim S-Y, Kim J-S, Oh J-S. The influence of gastrocnemius stretching combined with joint mobilization on weight-bearing ankle dorsiflexion passive range of motion. Journal of Physical Therapy Science. 2015;27(5):1317-8.

7. Kang M-H, Oh J-S, Kwon O-Y, Weon J-H, An D-H, Yoo W-G. Immediate combined effect of gastrocnemius stretching and sustained talocrural joint mobilization in individuals with limited ankle dorsiflexion: A randomized controlled trial. Manual Therapy. 2015;20(6):827-34.

8. Laudner K, Compton BD, McLoda TA, Walters CM. Acute effects of instrument assisted soft tissue mobilization for improving posterior shoulder range of motion in collegiate baseball players. International journal of sports physical therapy. 2014;9(1):1.

9. Carey-Loghmani M, Schrader J, Hammer W. Clinical foundations for graston technique® adapted from: graston technique® m1 instruction manual. 2014.
